# Characterization of the Small Exported *Plasmodium falciparum* Membrane Protein SEMP1

**DOI:** 10.1371/journal.pone.0103272

**Published:** 2014-07-25

**Authors:** Olivier Dietz, Sebastian Rusch, Françoise Brand, Esther Mundwiler-Pachlatko, Annette Gaida, Till Voss, Hans-Peter Beck

**Affiliations:** 1 Swiss Tropical and Public Health Institute, Department of Medical Parasitology and Infection Biology, Basel, Switzerland; 2 University of Basel, Basel, Switzerland; Institut national de la santé et de la recherche médicale - Institut Cochin, France

## Abstract

Survival and virulence of the human malaria parasite *Plasmodium falciparum* during the blood stage of infection critically depend on extensive host cell refurbishments mediated through export of numerous parasite proteins into the host cell. The parasite-derived membranous structures called Maurer's clefts (MC) play an important role in protein trafficking from the parasite to the red blood cell membrane. However, their specific function has yet to be determined. We identified and characterized a new MC membrane protein, termed small exported membrane protein 1 (SEMP1). Upon invasion it is exported into the RBC cytosol where it inserts into the MCs before it is partly translocated to the RBC membrane. Using conventional and conditional loss-of-function approaches we showed that SEMP1 is not essential for parasite survival, gametocytogenesis, or PfEMP1 export under culture conditions. Co-IP experiments identified several potential interaction partners, including REX1 and other membrane-associated proteins that were confirmed to co-localize with SEMP1 at MCs. Transcriptome analysis further showed that expression of a number of exported parasite proteins was up-regulated in SEMP1-depleted parasites. By using Co-IP and transcriptome analysis for functional characterization of an exported parasite protein we provide a new starting point for further detailed dissection and characterisation of MC-associated protein complexes.

## Introduction

The protozoan parasite *Plasmodium falciparum* causes the most severe form of human malaria responsible for nearly 700′000 deaths annually [1]. Its pathology is associated with the asexual development of the unicellular parasite within the red blood cell (RBC). Human RBCs are highly specialized cells devoid of all internal organelles hence survival of *P. falciparum* critically depends on extensive host cell refurbishments mediated by the export of parasite proteins into the RBC cytoplasm. This culminates in the insertion of the major parasite virulence factor PfEMP1 into the host cell membrane resulting in cell adhesion to receptors on endothelial cells. In that way, the parasite prevents elimination of infected RBCs in the spleen, but through this process causes pathology including organ failure and cerebral malaria [2].

Although protein export is the basis of all *P. falciparum* pathology, it is still not completely understood. It has been shown that many virulence proteins are exported via parasite derived membranous structures in the RBC cytoplasm termed Maurer's clefts (MCs), which concentrate virulence proteins for delivery to the RBC membrane [3], [4]. However, the role of MCs in this export of virulence proteins and their precise function remain elusive.

Many exported *P. falciparum* proteins contain a signal peptide (SP) and a PEXEL/VTS export motif [5], [6]. However, some of the best characterized MC proteins (MAHRP1, SBP1, REX1 and REX2) are PEXEL-negative exported proteins which do not contain a conserved export signal [7], [8], [9], [10]. While lacking a conserved export motif, these proteins share some similarities among each other: they are expressed early after invasion and mostly possess a transmembrane (TM) domain (MAHRP1, SBP1, REX2). Some PEXEL-negative proteins (e.g. REX1, MAHRP2) were shown to be membrane-associated rather than integral membrane proteins but still possess a predicted hydrophobic domain.

In our studies to elucidate the function and composition of MCs we identified and characterized a new MC-resident protein termed ‘small exported membrane protein 1′ (SEMP1: PF3D7_0702400/PF07_0007). SEMP1 is a small PEXEL-negative protein, expressed early during blood stage infection, and exported to the MCs before being partially translocated further to the RBC membrane where it is suggested to be involved in membrane modification.

## Results

### Subcelullar localization of SEMP1

**Figure 1:**
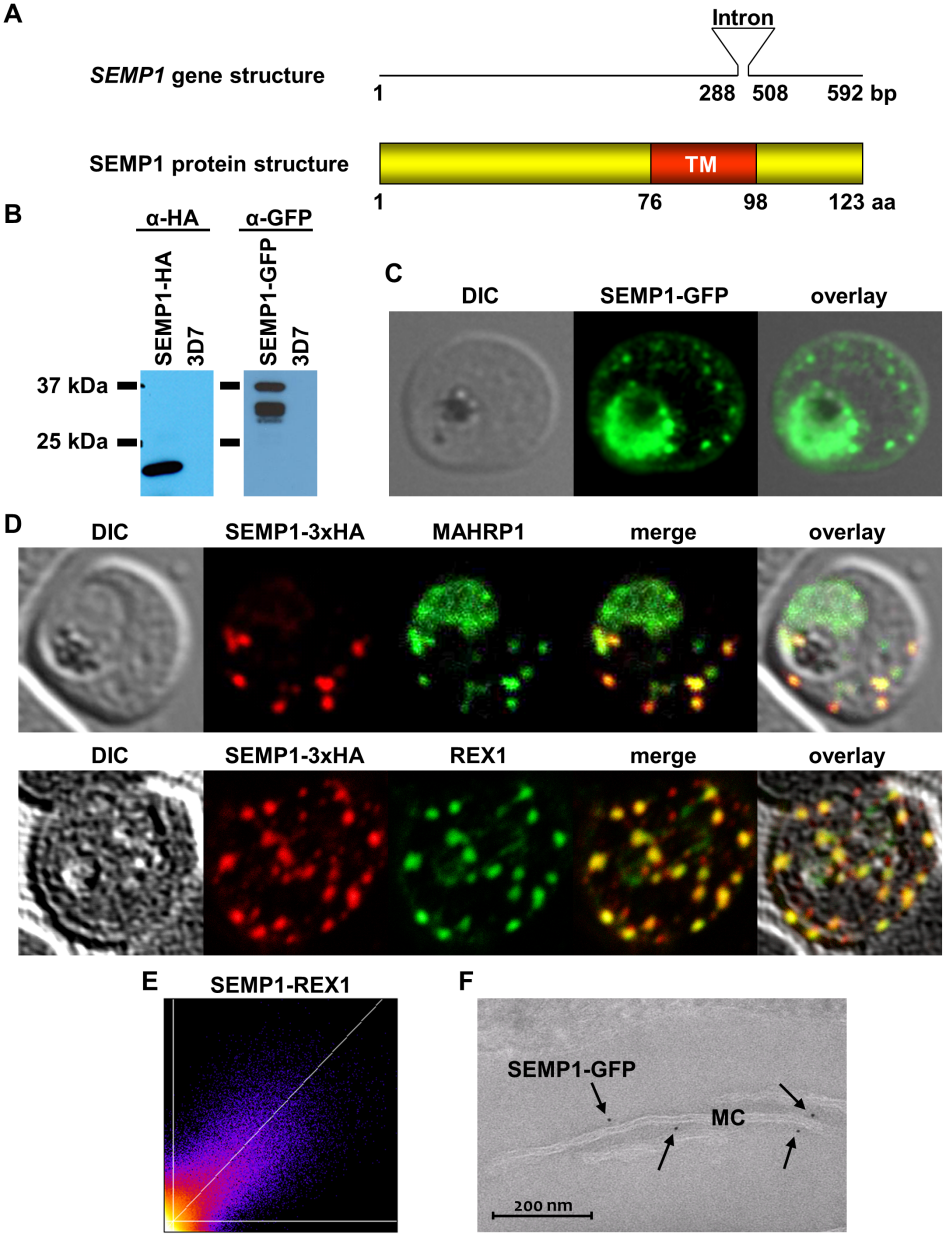
SEMP1 expression and localization. A: schematic representation of the *semp1* gene (top) and SEMP1 protein structure (bottom). TM, transmembrane protein. B: Live cell imaging of 3D7 parasites expressing SEMP1 with C-terminal tagged GFP. C: Immunofluorescence assays of MeOH-fixed RBCs infected with 3D7 expressing SEMP1 with a C-terminal 3xHA tag, co-labelled with rat α-HA and either rabbit α-MAHRP1 or rabbit α-REX1 antibodies. D: Scatter plot of co-localization of SEMP1 and REX1 in SEMP1-3xHA parasites. E: Electron microscopy (EM) of RBCs infected with 3D7 expressing SEMP1 with a C-terminal GFP tag labelled with rabbit α-GFP antibodies and decorated with 5 nm gold conjugated Protein A.

SEMP1 (PF3D7_0702400) is located on chromosome 7, contains one intron and encodes a protein of 123 amino acids (aa). This small, early expressed ‘*Plasmodium* protein with unknown function’ (PlasmoDB) has no PEXEL motif, no classical signal peptide but a single predicted transmembrane domain (TM) from aa 76 to 98 (Fig. 1A). Like many other PEXEL-negative exported proteins, no syntenic genes in other *Plasmodium* species exist but weak homologies to hypothetical proteins in *P. vixax, P. cynomolgi*, and *P. knowlesi* can be found.

We generated 3D7 transfectants expressing SEMP1 with a C-terminal green fluorescent protein (GFP) under the control of the PfCRT5' promoter using the transfection vector pARL [11]. Additionally, we also used a parasite line expressing SEMP1 C-terminally fused to a triple Hemagglutinin tag (3xHA) [12]. When parasite-lysates were probed with antibodies against 3xHA or GFP, a protein of approximately 20 kDa and 40 kDa in size was observed in SEMP1-3xHA and SEMP1-GFP transfectants (Fig 1B), respectively, and no signal was detected in uninfected RBCs.

**Figure 2:**
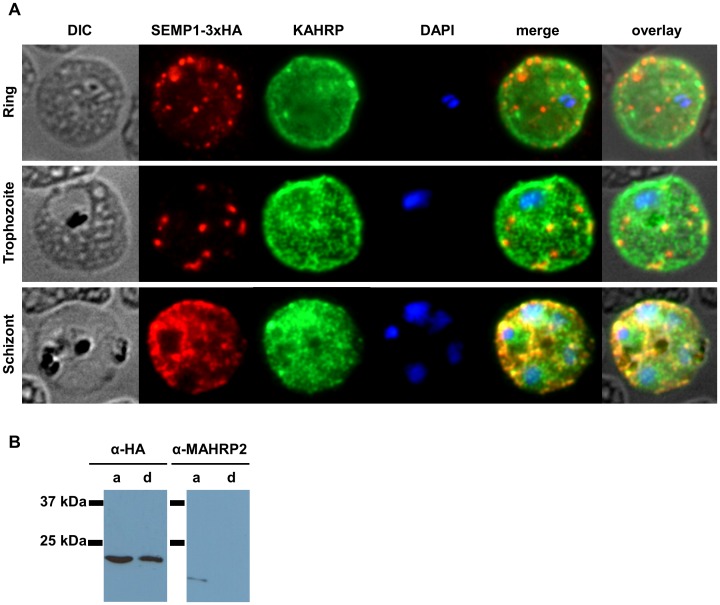
SEMP1 expression during blood stage development. A: Immunofluorescence assays of MeOH-fixed RBCs infected with 3D7 expressing SEMP1 with a C-terminal 3xHA tag, co-labelled with rat α-HA and mouse α-KAHRP antibodies. B: Parasite protein pellet of 3D7 expressing SEMP1-3xHA was fractionated using TritonX-114 into a soluble (aqueous phase) (a) and an insoluble (membrane) fraction (d). Proteins were visualized using mouse α-HA and rabbit α-MAHRP2 antibodies.

Live cell imaging of the SEMP1-GFP transfectants showed that the GFP-tagged protein was exported (Fig. 1C). There was also significant fluorescence observed within the parasite which might be due to protein overexpression. We further investigated the subcellular localization of SEMP1-3xHA by IFA using co-labelling with antibodies against the HA-tag and with either antibodies against MAHRP1 [13], REX1 [8], or KAHRP [14]. SEMP1-3xHA co-localized with MAHRP1 and REX1 to the MCs (Fig. 1D+1E). This confirms a study by Heiber and colleagues [15], which bioinformatically identified new PEXEL-negative proteins and also localized GFP-tagged SEMP1 to the MCs in ring stages and trophozoites. Electron microscopy using antibodies against GFP confirmed the localization of SEMP1-GFP at the MCs (Fig 1F). In later stages SEMP1-3xHA is further transported to the RBC membrane and partially localizes with KAHRP (Fig. 2A).

Solubility of SEMP1 was determined in Triton X114 where it was detected as an integral membrane protein but a substantial amount of protein was also found in the soluble fraction. In contrast, MAHRP2, a PEXEL-negative protein shown to be membrane-associated [16], was found exclusively in the soluble protein fraction confirming that the membrane fraction was not contaminated with soluble proteins (Fig. 2B).

**Figure 3:**
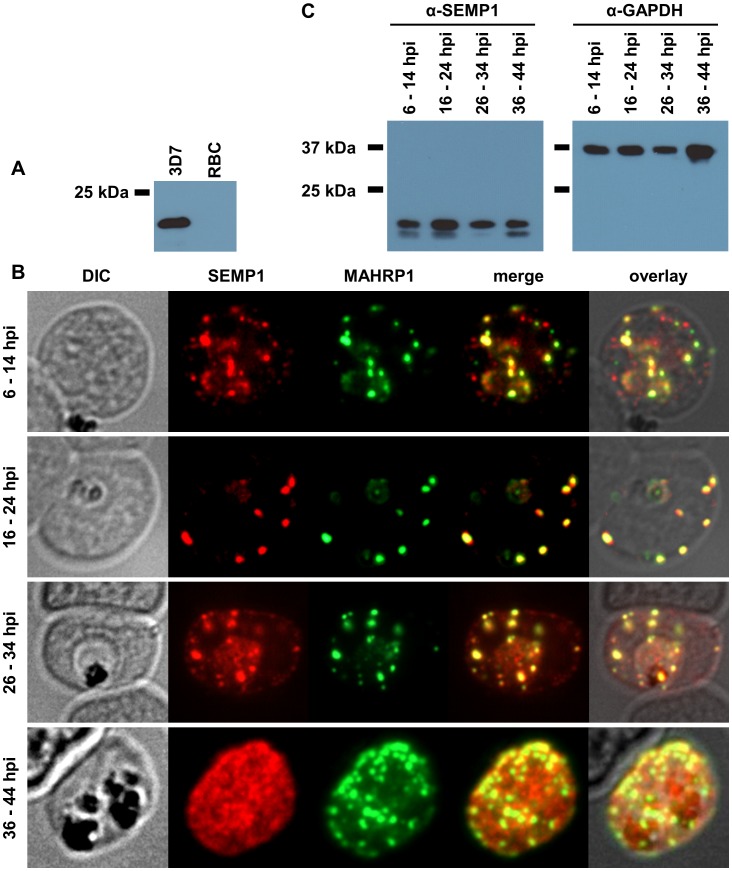
Localization and expression of endogenous SEMP1 in 3D7 wild-type parasites. A: 3D7 wild-type parasite lysate (3D7) and uninfected red blood cells (RBC) were analysed by immunoblotting and probed with mouse serum raised against recombinant full-length SEMP1. B: IFAs of MeOH-fixed RBCs infected with 3D7 wild-type parasites synchronized at timepoints 6–14 hours post invasion (hpi), 16–24 hpi, 26–34 hpi and 36–44 hpi. Cells were co-labelled with mouse α-SEMP1 and rabbit α-MAHRP1 serum. C: 3D7 parasite pellets were generated at the respective timepoints by saponin lysis and analysed by immunoblot using mouse α-SEMP1 serum and mouse α-GAPDH (PF3D7_1462800) antibodies.

To localize endogenous SEMP1, we generated affinity purified mouse antibodies raised against recombinant full-length SEMP1 protein. These antibodies recognized a protein of expected 15 kDa in 3D7 parasite lysates but not in uninfected RBCs (Fig. 3A). Dual-labelling IFAs of sorbitol-synchronized parasites at 6–14 hours post invasion (hpi), 16–24 hpi, 26–34 hpi, and 36–44 hpi showed that SEMP1, similarly to MAHRP1, is exported early after invasion to MCs where it partially co-localized with MAHRP1 (Fig 3B). In younger stages SEMP1 was not found at all MCs whereas in later stages it is transported further to the RBC membrane. Western blots with parasite lysates taken at corresponding time points showed that endogenous SEMP1 is constantly expressed throughout the asexual intra-erythrocytic cycle (Fig 3C).

### Requirements for export of SEMP1

**Figure 4:**
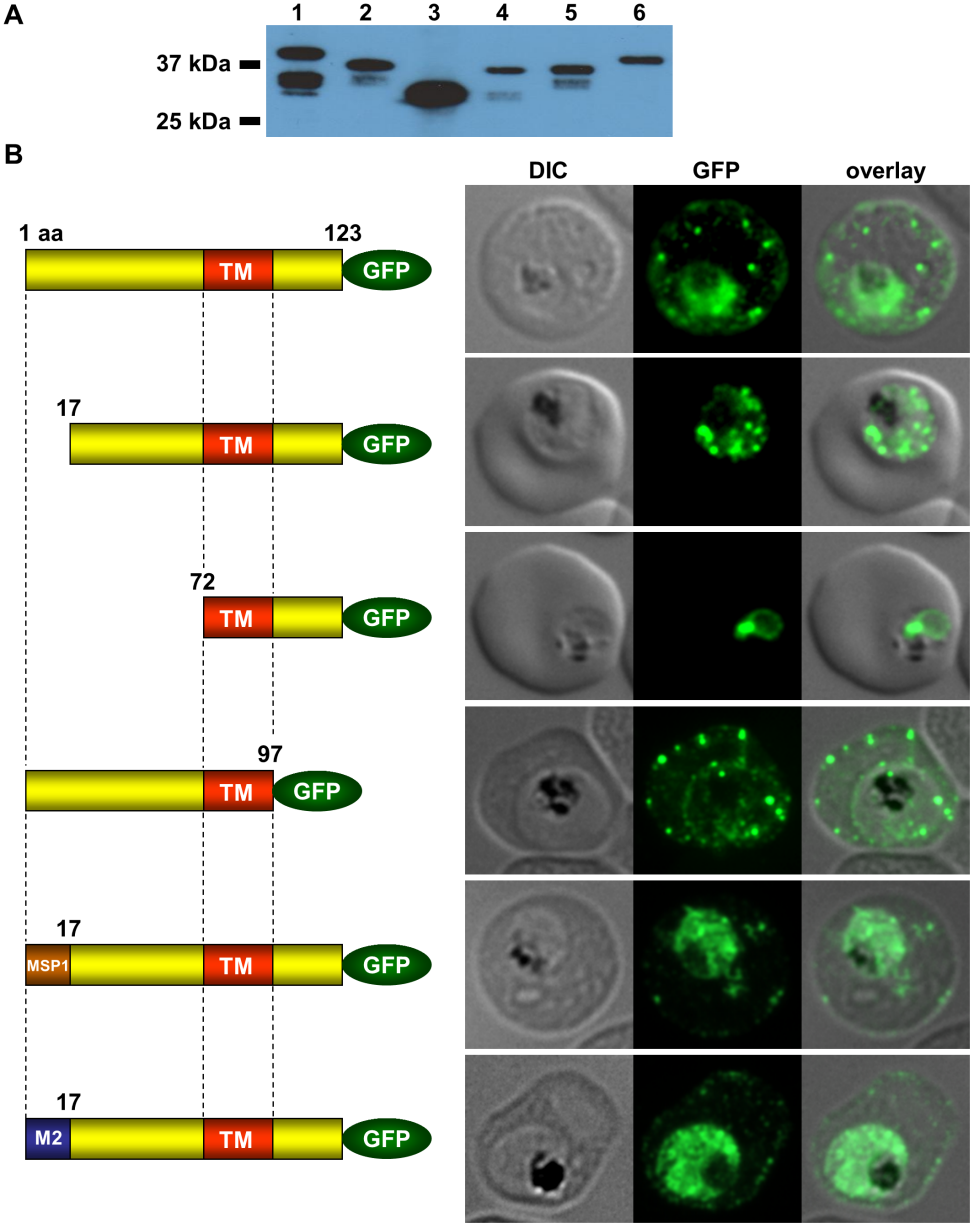
Requirements for SEMP1 export into the RBC. A: Lysates of SEMP1-KO parasites expressing full-length and truncated or mutated forms of SEMP1 C-terminally fused to GFP were generated by saponin lysis and analysed by immunoblotting using mouse α-GFP antibodies. Lane 1: SEMP1_1–123_-GFP (full-length), lane 2: SEMP1_17–123_-GFP, lane3: SEMP1_72–123_GFP, lane4: SEMP1_1–97_-GFP, lane 5: MSP1_1–16_SEMP1_17–123_-GFP, lane 6: MAHRP2_1–16_SEMP1_17–123_-GFP. B: Immunofluorescence assays of MeOH-fixed RBCs infected with SEMP1-KO parasites expressing full-length and truncated or mutated forms of SEMP1 C-terminally fused to GFP. Expressed SEMP1 was labelled with rabbit α-GFP antibodies. The transmembrane domain is depicted in red (TM), a MSP1 signal peptide in brown (MSP1) and the MAHRP2 N-terminus in blue (M2).

To elucidate sequence requirements for SEMP1 export SEMP1-KO parasites (see below) were complemented with plasmids expressing full-length, truncated, or mutated versions of SEMP1 with a C-terminal GFP-tag (Fig. 4A). SEMP1-KO parasites were used to avoid any interference with endogenous protein. IFAs showed that deletion of the complete C-terminus of SEMP1 (SEMP1_1–97_-GFP) did not impair export and resulted in correct localisation whilst deletion of the first 16 amino acids (SEMP1_17–123_-GFP) of the N-terminus resulted in an impaired export from the parasite. The deletion of 71 amino acids (SEMP1_72–123_-GFP) of the N-terminus prevented export and seemed to have resulted in concentration of SEMP1 in or at the endoplasmic reticulum (Fig. 4B). If the first 16 amino acids of SEMP1 were replaced by the corresponding amino acids of MAHRP2 (M2_1–16_SEMP1_17–123_-GFP) or the classical signal peptide of MSP1 (MSP1_1–16_SEMP1_17–123_-GFP) the fusion protein was exported in both cases albeit with lower efficiency (Fig. 4B).

### Identification of potential SEMP1 interaction partners by Co-IP

**Figure 5:**
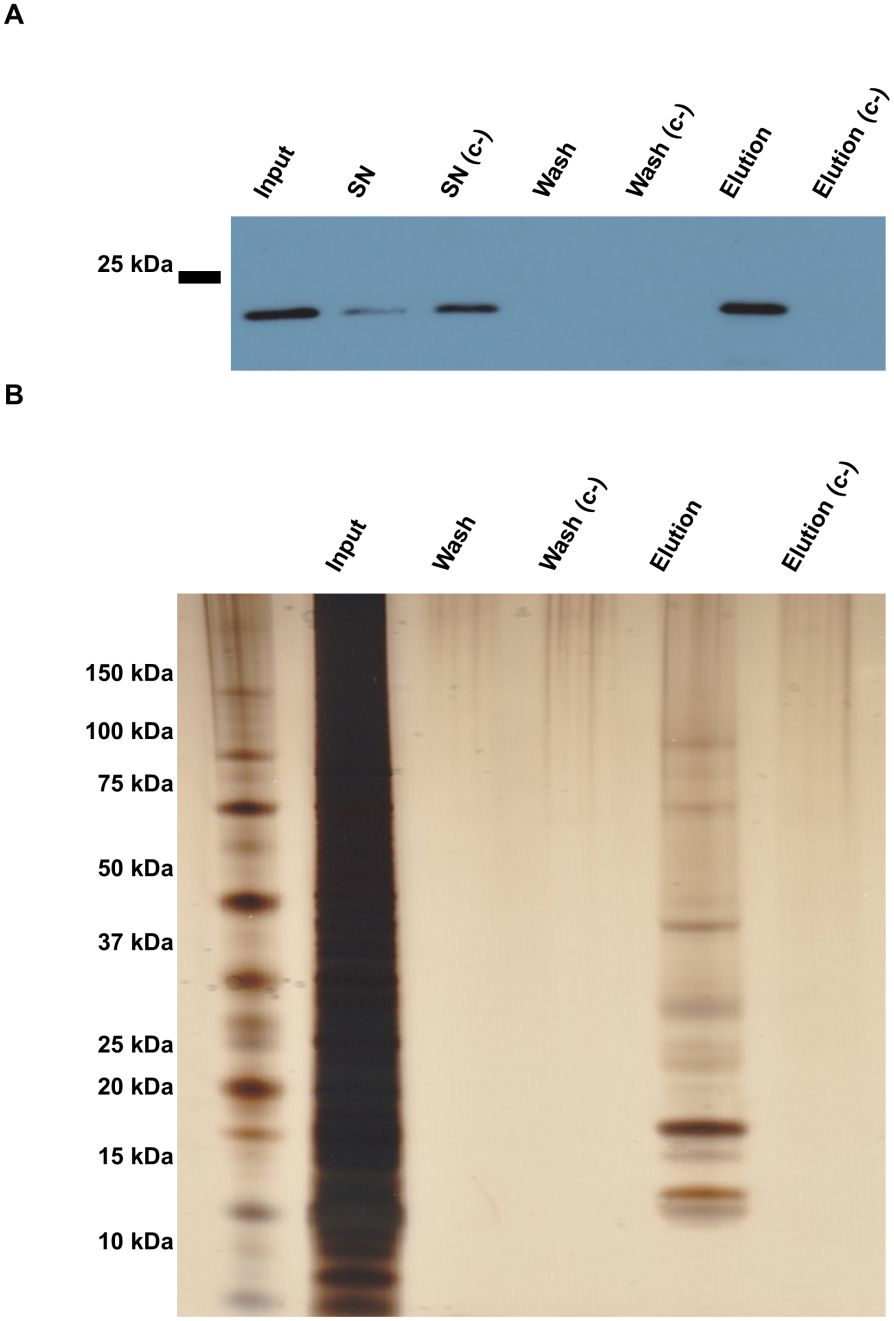
Identification of potential SEMP1 interaction partners by Co-IP. Co-IP was performed with 3D7 parasites expressing SEMP1 with a C-terminal 3xHA tag. Cultures were cross-linked with 1% formaldehyde and parasites were released by saponin treatment, lysed in 1%SDS followed by sonication. Lysate (Input) was incubated with α-HA affinity matrix. After centrifugation the matrix was washed three times with washing buffer (Wash) and proteins were eluted from by HA peptide elution. As a negative control, an excess of soluble HA peptides was added to the input to block the HA binding sites of the matrix (c-). Samples were analysed by Western blot with α-HA antibodies (A) and by silver staining (B). Proteins co-eluted with SEMP1-3xHA were identified by MS analysis of both TCA precipitated total elution (precipitation) and Coomassie-stained gel slices (gel extraction).

To reveal proteins interacting with SEMP1 we performed co-immunoprecipitation (Co-IP) experiments followed by mass spectrometry-based protein identification. Using protein extracts of trophozoite stage SEMP1-3xHA parasites, potential interacting proteins were eluted from the HA-affinity matrix by competition with soluble HA peptides. A protein extracts to which an excess of HA peptides was added during the binding step was used as negative control. Western blot analysis confirmed that SEMP1-3xHA was successfully purified and eluted (Fig. 5A), and silver staining revealed that additional proteins were co-eluted with SEMP1-3xHA (Fig. 5B). Table 1 summarizes the most probable interaction partners for which ≥5 peptides were detected in LC-MS/MS analysis of either TCA precipitated elution or from Coomassie-stained SDS-PAGE gel slices.

**Table 1:** Identification of potential SEMP1 interaction partners by Co-IP and MS analysis.

Gene ID	Gene product	N (peptides) elution	N (peptides) elution (c-)	N (peptides) Gel extraction	localization
PF3D7_0702400	SEMP1	**12**	0	**67**	MC
PF3D7_0935900 *	REX1	**5**	0	**29**	MC
PF3D7_0702500 *	Plasmodium exported protein	**6**	0	**19**	MC
PF3D7_0601900 *	Conserved Plasmodium protein	**5**	0	**13**	MC
PF3D7_0831700	PfHsp70-x	0	0	31	J-dots
PF3D7_0818900	HSP70	0	0	15	no
PF3D7_0708400	HSP90	0	0	15	no
PF3D7_1353200	MAHRP2	0	0	12	tethers
PF3D7_1149000 *	Pf332	0	0	11	MC
PF3D7_1016300	Glycophorin Binding Protein GBP	0	0	11	RBC
PF3D7_0507100	60S ribosomal protein L4	0	0	9	no
PF3D7_0517000	60S ribosomal protein L12	0	0	6	no
PF3D7_0501200 *	PIESP2	0	0	5	MC
PF3D7_1462800	GAPDH	0	0	5	no

localization during asexual intra-erythrocytic cycle: MC  =  Maurer's clefts; RBC  =  soluble in RBC cytosol, no  =  not exported. Candidates investigated for co-localization with SEMP1 are marked by an asterisk.

To confirm potential interacting partners 3D7 parasites were transfected with constructs to express candidate proteins with either a C-terminal 3xHA tag (PF3D7_0702500/PF3D7_0601900), or with a GFP tag (PIESP2)(Fig S1).

**Figure 6:**
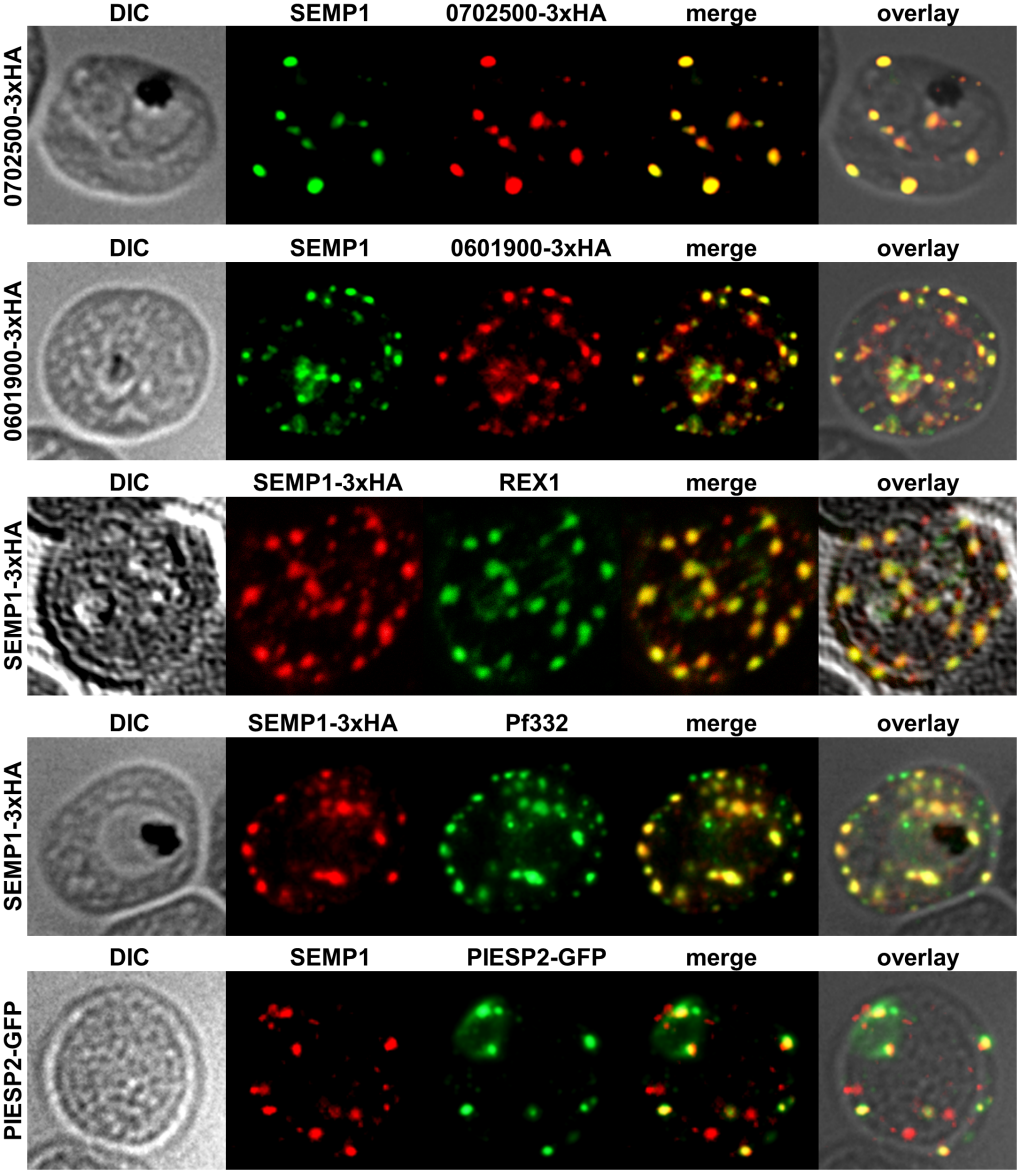
Localization of potential SEMP1 interacting proteins. Immunofluorescence assays of MeOH-fixed RBCs infected with 3D7 expressing SEMP1/PF3D7_0702500/PF3D7_0601900 with a C-terminal 3xHA tag and 3D7 expressing PIESP2 with a C-terminal GFP-tag, co-labelled with mouse α-SEMP1 and rat α-HA (PF3D7_0702500-3xHA & PF3D7_0601900-3xHA) /α-GFP (PIESP2-GFP). For co-labelling of SEMP1-3xHA with REX1 and Pf332, rat α-HA and rabbit α-REX1 / mouse α-Pf332 antibodies were used.

We determined the subcellular localization of these potential interaction partners by IFAs using mouse anti-SEMP1 antibodies combined with either rat anti-HA antibody for PF3D7_0702500-3xHA and PF3D7_0601900-3xHA or rabbit anti-GFP antibody for PIESP2-GFP. For Pf332 we used mouse anti-Pf332 antibody and rat anti-HA antibody in parasites expressing SEMP1_3xHA. REX1 co-localization was determined using rabbit anti-REX1 antibodies and mouse anti-SEMP1 antibodies. All potential interaction partners co-localized at least partially with SEMP1, but in particular with Pf332 or PIESP2 antibodies only a fraction of SEMP1 positive MCs were labelled with either antibody suggesting that distinct subpopulations of MCs exist (Fig. 6).

### Knockout of SEMP1

**Figure 7:**
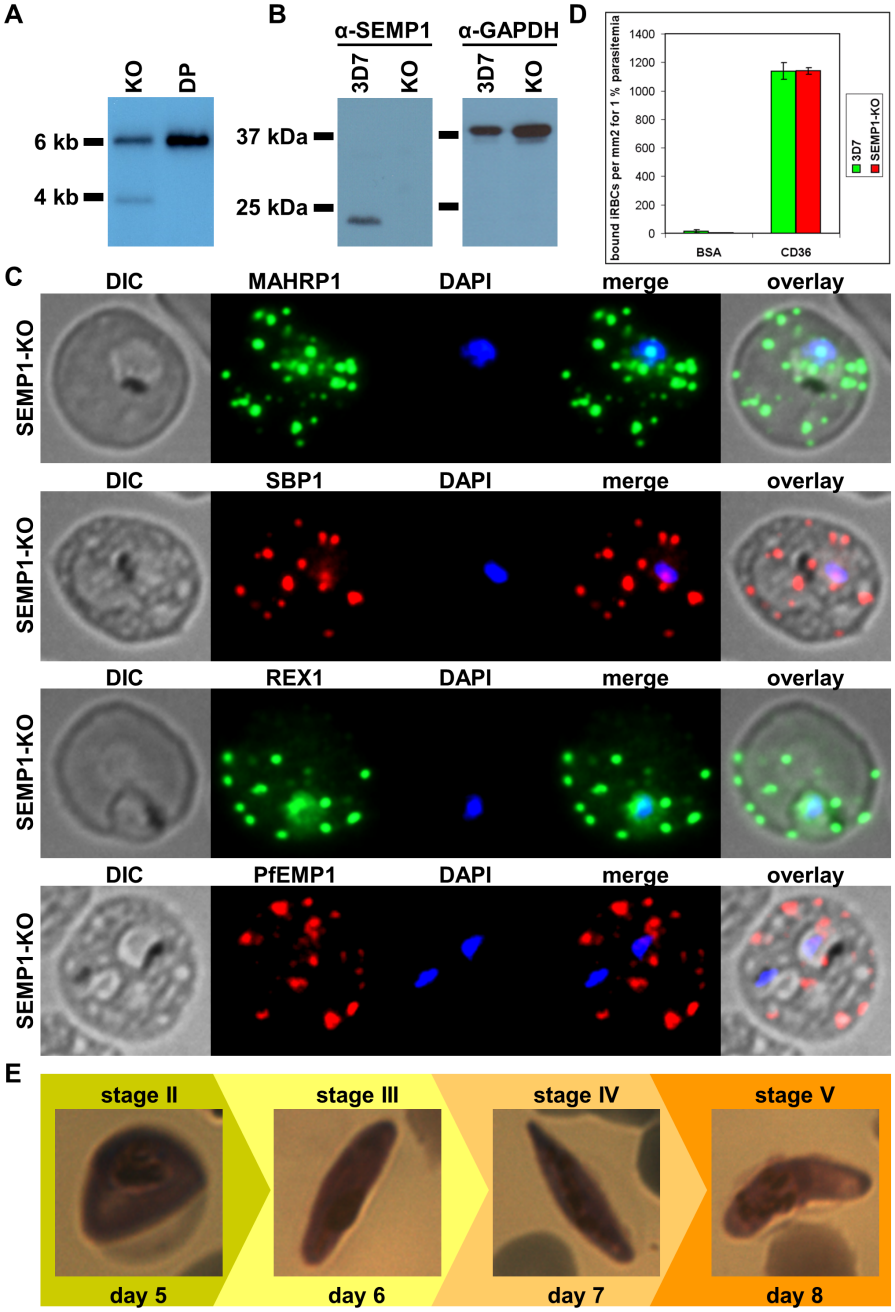
Knockout of SEMP1 by gene disruption has no detectable phenotype. A: *Cla*I- and *Xma*I-digested gDNA isolated from a SEMP1-KO clone (KO) pH-KO plasmid (DP) were analysed by Southern blot and probed with radioactively labelled *hdhfr*. In case of an integration of the KO plasmid into the parasite genome the expected fragment size is 3927 bp. B: lysates of 3D7 wild type and SEMP1-KO (KO) parasites were generated by saponin lysis and analysed by Western blot with α-SEMP1 and α-GAPDH (loading control) antibodies. C: Immunofluorescence assays of fixed RBCs infected with SEMP1-KO parasites, probed with α-MAHRP1, α-SBP1, α-REX1 and α-PfEMP1 antibodies. The parasite nuclei were stained with DAPI. D: Bar graph depicting differential binding to CD36 of RBCs infected with wild-type 3D7 and SEMP1-KO parasites. CD36 (or BSA as control) was immobilized on Petri dishes and bound infected RBCs (iRBCs) were calculated as iRBCs per mm^2^ for 1% parasitemia.

To investigate the function of SEMP1, the gene was knocked out in 3D7 wild type parasites by single crossover gene disruption. Integration of the human dihydrofolate reductase (*hdhfr*) cassette-containing plasmid pH-SEMP1-KO into the correct location of the parasite genome was confirmed by Southern blot (Fig. 7A) and a parasite clone was isolated by serial dilution. Western blot analysis using polyclonal antibodies against SEMP1 showed that the gene disruption was successful and SEMP1 was absent (Fig 7B). SEMP1-KO parasites were viable in absence of SEMP1 and also showed no adverse growth effects in culture.

To test if transport of other proteins was disturbed we analysed the SEMP1-KO parasite clone by IFA using antibodies against MAHRP1, SBP1, REX1 and PfEMP1. All four tested proteins were still exported in absence of SEMP1 (Fig. 7C). We further tested whether PfEMP1 was correctly exposed on the RBC surface of KO parasites by testing binding of iRBCs to CD36 under static conditions but no differences were observed when compared to the parental parasites (Fig. 7D). Lastly, we tested whether gametocytogenesis could be induced in the SEMP1-KO strain and after five days type II gametocytes appeared and fully matured to stage V gametocytes similarly as in the wild type (Fig. 7E).

**Figure 8:**
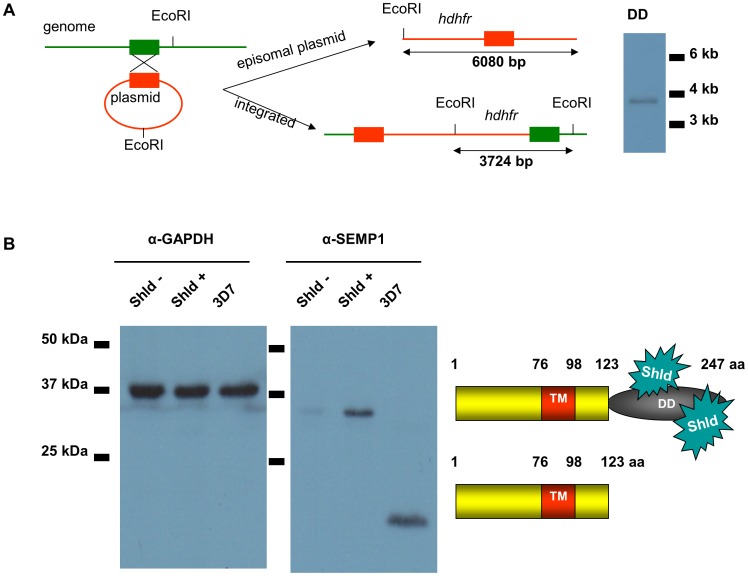
Conditional Knockdown of SEMP1. A: *EcoR*I-digested gDNA isolated from SEMP1-DD parasites (DD) was analysed by Southern blot and probed with radioactively labelled *hdhfr* B: A culture of 3D7 parasites expressing SEMP1-DD was split and cultured for 2 weeks in presence (Shld +) or absence (Shld -) of the small molecule Shield-1. Whole parasite-lysates were generated after saponin lysis and analysed by Western blot using α-SEMP1. As a loading control the blot was additionally probed with antibodies against the housekeeping protein GAPDH.

To have the ability to investigate the SEMP1 function under controlled conditions and between isogenic populations we also generated a conditional SEMP1 knockdown parasite in 3D7 by fusing a FKBP destabilization domain (DD) to the C-terminus of the endogenous SEMP1. DD confers instability to the fusion protein resulting in its targeted degradation if not stabilised by the small molecule Shield-1 [17], [18]. Integration of the DD and *hdhfr* cassette-containing plasmid pARL-SEMP1-DD at the correct locus was confirmed by Southern blot (Fig. 8A). Western blot analysis of synchronized SEMP1-DD parasites grown for 2 weeks without Shield showed significantly reduced amounts SEMP1 and only minute amounts of protein could be detected (Fig. 8B).

### Transcriptional changes in absence of SEMP1

To test if parasites responded to SEMP1 depletion with possible compensatory changes in gene expression we conducted comparative transcriptional profiling using genome-wide microarray technology. Parasite RNA was isolated at four specific time points (6–14 hpi, 16–24 hpi, 26–34 hpi & 36–44 hpi) from synchronized SEMP1-DD parasites cultured with and without Shield. The Cy5-labelled SEMP1-DD cRNA was mixed with an equal amount of Cy3-labelled 3D7 reference cRNA (mixed stages) and hybridized onto an Agilent *P. falciparum* Microarray slide (AMADID #037237) containing oligonucleotides for all *P. falciparum* genes [19]. We did not detect any significant transcriptional changes in SEMP1-depleted parasites using Significance Analysis for Microarrays [20]. However, using the less stringent Student's t-test, a total of 34 genes were significantly (p<0.05) up-regulated over either two, three or four consecutive time points with a relative average fold change (RAFC) >1.5. Down-regulation was observed for twelve parasite genes with a RAFC <1.5 (p<0.05). The highest fold change of 2.18 was seen for a *var* gene (PF3D7_0900100) between 6 and 14 hours post invasion (hpi). The most highly down-regulated gene codes for a conserved protein with unknown function (PF3D7_1475200) with a 2-fold change between 6 and 14 hpi (Table 2).

**Table 2:** Transcriptional changes in absence of SEMP1 (microarray data).

GeneID	FC 1	FC 2	FC 3	FC 4	RAFC	Product Description
**PF3D7_0900100**	**2.18**	**1.26**	**1.08**	**2.00**	**2.09**	**PfEMP1**
**PF3D7_1301400**	**0.79**	**1.77**	**1.84**	**2.02**	**1.93**	**P. exported protein (hyp12)**
**PF3D7_0532300**	**0.74**	**2.15**	**1.56**	**1.59**	**1.77**	**P. exported protein (PHISTb)**
**PF3D7_0201900**	**0.56**	**1.71**	**1.69**	**1.27**	**1.70**	**PfEMP3**
PF3D7_0618200	1.15	1.08	1.75	1.63	1.69	conserved Plasmodium protein
PF3D7_0903700	0.95	0.70	1.62	1.72	1.67	alpha tubulin 1
PF3D7_1468200	1.00	1.41	1.60	1.70	1.65	conserved protein,
**PF3D7_0831700**	**1.02**	**1.27**	**1.62**	**1.66**	**1.64**	**heat shock protein 70 (HSP70-x)**
PF3D7_0113000	0.71	1.60	1.65	1.62	1.64	glutamic acid-rich protein (GARP)
PF3D7_0319900	1.63	1.38	1.16	1.61	1.62	conserved Plasmodium protein,
PF3D7_0821700	1.15	1.47	1.47	1.91	1.61	60S ribosomal protein L22
PF3D7_1226300	1.83	1.36	1.62	1.24	1.60	cof-like hydrolase
PF3D7_1415700	1.21	1.62	1.53	1.42	1.57	serine C-palmitoyltransferase
PF3D7_0804800	1.55	1.10	1.57	1.57	1.57	peptidyl-prolyl isomerase (CYP24)
**PF3D7_1200600**	**1.41**	**1.49**	**1.79**	**0.89**	**1.56**	**PfEMP1 (VAR2CSA)**
PF3D7_1242700	0.97	1.19	1.56	1.57	1.56	40S ribosomal protein S17
PF3D7_0629100	1.56	0.74	1.25	1.56	1.56	phosphoribosyltransferase
**PF3D7_0936800**	**0.85**	**1.57**	**1.38**	**1.71**	**1.55**	**P. exported protein (PHISTc)**
PF3D7_0323000	1.52	0.87	1.39	1.59	1.55	conserved Plasmodium protein
PF3D7_1317900	0.87	1.53	1.58	1.38	1.55	nuclear export protein
**PF3D7_1476200**	**1.22**	**1.42**	**1.84**	**1.38**	**1.55**	**P. exported protein (PHISTb)**
PF3D7_0210900	1.11	1.04	1.49	1.58	1.54	conserved Plasmodium protein
PF3D7_0403700	1.23	1.11	1.52	1.55	1.53	CGI-201 protein, short form
PF3D7_0218100	1.70	1.47	0.80	1.43	1.53	conserved P. membrane protein
PF3D7_1138500	1.53	1.51	1.16	1.36	1.52	protein phosphatase 2c
**PF3D7_0402000**	**0.96**	**0.85**	**1.47**	**1.56**	**1.52**	**P. exported protein (PHISTa)**
PF3D7_1305400	1.53	1.38	1.65	1.16	1.52	conserved Plasmodium protein
PF3D7_1474000	1.50	1.52	1.07	0.95	1.51	probable protein
PF3D7_0708800	1.04	0.84	1.49	1.53	1.51	heat shock protein 70 (HSP70-z)
**PF3D7_1353100**	**1.07**	**1.45**	**1.50**	**1.51**	**1.51**	**Plasmodium exported protein,**
PF3D7_1426600	1.55	1.28	1.69	1.24	1.51	conserved Plasmodium protein
PF3D7_1443900	1.09	0.77	1.49	1.52	1.51	heat shock protein 90 (HSP90)
PF3D7_1011800	1.42	0.99	1.64	1.46	1.51	QF122 antigen
PF3D7_1237900	1.24	1.51	1.50	0.50	1.50	conserved Plasmodium protein
**PF3D7_0113400**	**0.60**	**0.79**	**1.34**	**0.60**	**0.60**	**Plasmodium exported protein**
PF3D7_0930300	1.26	1.14	0.61	0.58	0.59	merozoite surface protein 1
PF3D7_0503200	0.59	0.60	0.70	1.30	0.59	conserved Plasmodium protein
PF3D7_0827600	0.60	0.56	1.08	1.55	0.58	conserved Plasmodium protein
PF3D7_1231300	0.50	0.60	0.61	0.60	0.57	conserved Plasmodium protein
PF3D7_1216500	0.58	0.55	0.84	1.08	0.56	male development gene 1 (MDV1)
PF3D7_0302200	1.01	1.04	0.57	0.56	0.56	cytoadherence linked protein 3.2
PF3D7_0728800	0.57	0.55	1.25	1.55	0.56	conserved Plasmodium protein
PF3D7_0217500	0.79	1.12	0.56	0.54	0.55	calcium dependent protein kinase 1
PF3D7_1307500	0.56	0.78	0.93	0.52	0.54	conserved Plasmodium protein
PF3D7_1112200	0.54	0.53	0.88	1.34	0.54	coq4 homolog, putative
PF3D7_1475200	0.50	0.54	0.83	1.28	0.52	conserved protein

Summary of all significantly (p<0.05) up- (top) and down-regulated (bottom) parasite genes with a respective average fold change (RAFC) >1.5 or <0.6. The RAFC is thereby the average FC over the significantly up-/down-regulated time points only. The time points were 6–14 hpi (FC1), 16–24 hpi (FC2), 26–34 hpi (FC3) and 36–44 hpi (FC4). Exported parasite proteins are highlighted in bold.

**Figure 9:**
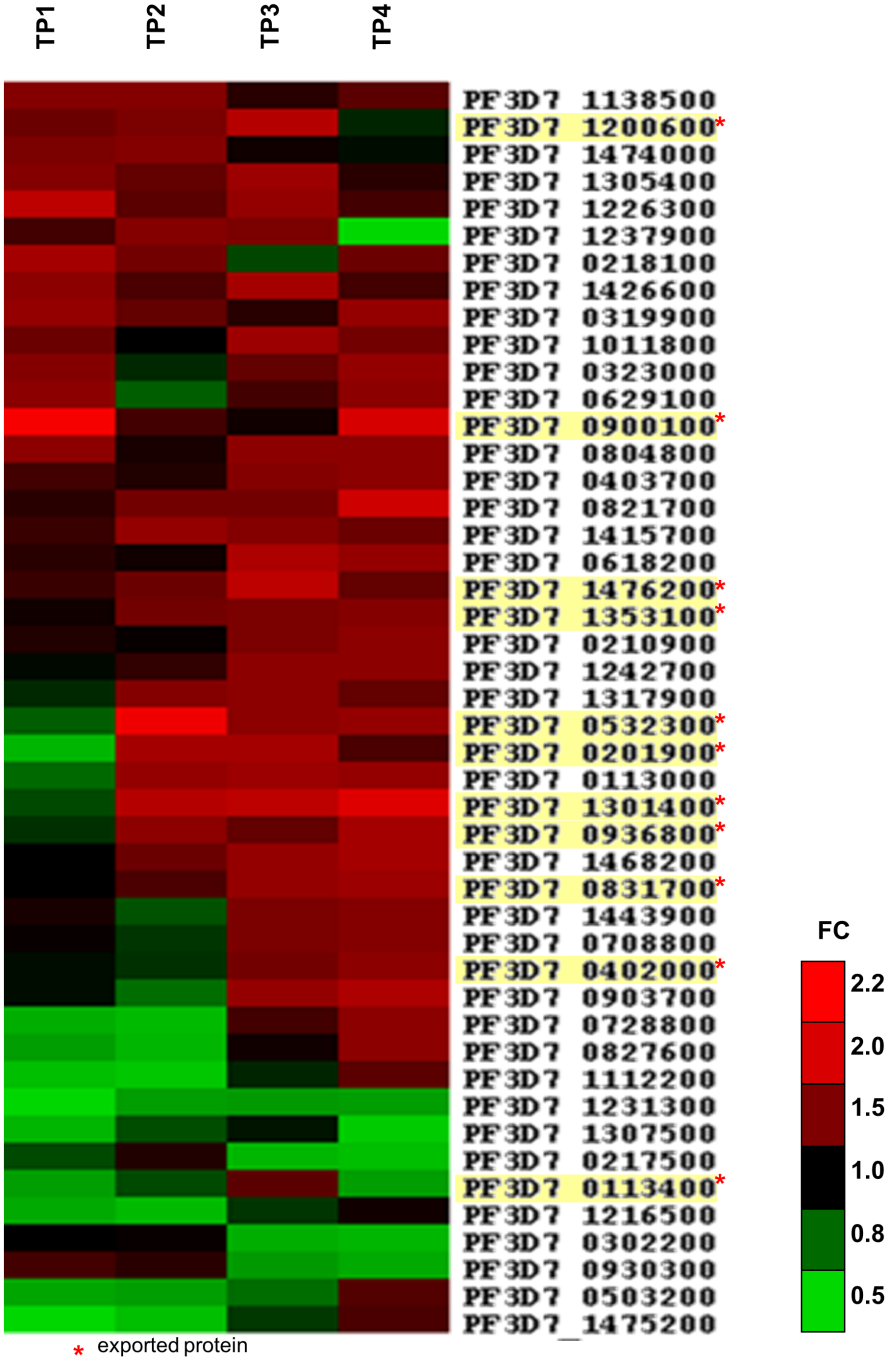
Transcriptional changes in absence of SEMP1 identified by microarray analysis. Summary of all significantly (p<0.05) up- and down-regulated parasite genes with a respective average fold change (RAFC) >1.5 or <0.6. The RAFC is thereby the average FC over the significantly up- / down-regulated time points (TPs) only. A graphic depiction of their FCs throughout the four time points (TPs) is shown in form of a heat map. Up-regulation (FC>1) is indicated in red, down-regulation (FC<1) in green. The time points were 6–14 hpi (TP1), 16–24 hpi (TP2), 26–34 hpi (TP3) and 36–44 hpi (TP4). Exported proteins are highlighted in yellow.

A total ten of the up-regulated genes, including the four most highly up-regulated genes, encode known exported proteins (Table 2), namely PfEMP1, HYP12, PHISTb and PfEMP3. In contrast, only one of the twelve down-regulated genes codes for a predicted exported protein with unknown function (PF3D7_0113400). Figure 9 shows that up-regulation of the exported proteins did mostly not peak after invasion but during the trophozoite and schizont stage.

Strikingly, two of the ten up-regulated exported proteins were also identified as potential interaction partners of SEMP1: HSP70-x (PF3D7_0831700) and a protein with unknown function (PF3D7_1353100). Although all other proteins identified as potential SEMP1 interaction partners did not have a RAFC >1.5, four of them were still significantly (p<0.05) up-regulated: REX1 (RAFC  = 1.20), PF3D7_0702500 (RAFC  = 1.33), PF3D7_0601900 (RAFC  = 1.35) and GBP (RAFC  = 1.44).

## Discussion

MCs have a crucial role in protein trafficking to the iRBC membrane and there is evidence that they act as secretory organelles concentrating virulence proteins destined for the host cell membrane [3], [4], but further functions have yet to be determined. Similarly, the protein composition of MCs is not well established and there are steadily new proteins revealed, which are either resident or transiently located to the MCs indicating that the current MC proteome is far from complete. Since many of the MC proteins lack a signal peptide (SP) and a PEXEL/VTS motif it impossible to virtually establish a comprehensive MC proteome.

We Identified and characterized a new MC protein termed SEMP1 that is a small protein with unknown function. It has neither a PEXEL-motif nor a SP (PlasmoDB) and the SMART protein domain prediction tool [21] identified a single transmembrane (TM) domain from amino acid 76 to 98 (Fig. 1A). Similar to the MC resident proteins MAHRP1 and SBP1, it is expressed early and exported to the MCs where it integrates into the MC membrane. However, unlike MAHRP1 or SBP1 which persist as integral MC membrane proteins throughout the whole intra-erythrocytic cycle [13], [22] SEMP1 is translocated further to the RBC membrane in schizonts (Fig. 3B).

Functional information is available for only few MC proteins, e.g. REX1 null mutants showed export of PfEMP1 to the MCs but subsequently was not efficiently presented at the RBC surface [23) whilst MAHRP1 or SBP1 knock-out parasites showed a lack of PfEMP1 translocation [9], [24], [25]. To study the role of SEMP1 in protein export we generated a SEMP1 knock-out parasite by single crossover gene disruption and also generated a conditional SEMP1 knock down parasite by fusing the FKBP destabilization domain (DD) to the C-terminus of endogenous SEMP1. Both parasite lines showed no obvious phenotypic change under *in vitro* culture conditions. All tested proteins (REX1, SBP1, MAHRP1, and PfEMP1) were trafficked to their correct destinations in SEMP1-KO parasites, there was no growth reduction, and gametocyte production was similar to wild type parasites suggesting that SEMP1 is not essential for PfEMP1 export or gametocytogenesis. This indicates redundancy of functional pathways in which other proteins might compensate for the loss of SEMP1, or that SEMP1 may have other functions during the intraerythrocytic cycle which escaped our observation such as modifying fitness in natural human infections.

We used Co-IP to identify potential interaction partners of SEMP1 and in two independent experiments 13 different *P. falciparum* proteins were found of which 3 were identified in both experiments, namely REX1, PF3D7_0702500, and PF3D7_0601900. While REX1 is a known MC resident protein, both other proteins were uncharacterized. PF3D7_0702500 and PF3D7_0601900 have similarities to *semp1* in that they have one intron, are non-syntenic to other *Plasmodium* species and code for small parasite proteins with unknown function (PlasmoDB). They also have a predicted single TM domain and no PEXEL/VTS motif or signal peptide. Their localization to the MCs was recently confirmed in a bioinformatics study which identified new PEXEL-negative exported proteins [15]. Since parasite proteins were extracted with 1% sodium dodecyl sulphate (SDS), a detergent that emulsifies membrane lipids and proteins, it is conceivable these MC transmembrane proteins were co-precipitated as constituents of the same complex and not just as constituents of the same membrane fragment. None of the other identified exported proteins yet has been shown to be resident in the MCs. PfHsp70-x is an exported chaperone which in a complex with co-chaperones forms highly mobile structures in the RBC cytosol called J-dots [26], [27]. MAHRP2 has been described as a PEXEL-negative protein forming cylindrical structures called tethers which are believed to tether the MCs to the RBC skeleton [16] and Pf332 is a large *P. falciparum* protein exported into the RBC [28], [29] where it similar to SEMP1 closely associates with MCs [30], [31] but also partly with the RBC membrane [28], [30], [32]. The parasite-infected erythrocyte surface protein 2 (PIESP2) has been identified as a TM protein in the MCs [33] but might be partly associated with the RBC membrane [34]. This is one of the first studies using Co-IP and MS/MS to identify interaction partners of exported *P. falciparum* proteins which still need further confirmation but the remarkable cleanliness achieved through HA peptide elution suggests promising interaction candidates.

Since no phenotype was observed upon knocking out SEMP1, we speculated that lack of SEMP1 might have been compensated for by altered expression of other genes. This was monitored by a comparative transcriptome analysis with SEMP1-DD parasites grown with or without Shield-1 allowing the comparison of genetically identical parasites at four consecutive time points. We identified 34 genes that were significantly up-regulated over at least two consecutive time points and the 4 highest RAFC were found with exported proteins. Up-regulation of a *var* gene (PF3D7_0900100) was surprising since *var* genes regularly switch from expression of one *var* gene to another. In contrast, In contrast, PF3D7_1301400 codes for an early expressed predicted exported protein with SP and PEXEL motif belonging to the hypothetical gene family *hyp12*. There are 3 *hyp12* paralogs with no orthologs in any other organism not possessing any annotated protein domains making them elusive for functional predictions [35]. However, other HYP proteins have been shown to be variantly expressed [36] and it might be speculated that SEMP1 knockdown parasites switched to expression of another HYP12. This might similarly apply to PF3D7_0532300 which is a member of the PHIST family for which also variant transcriptional has been shown [36] but its function is unknown. PHIST proteins have been shown to be involved in many functions such as modification of the infected host cell, alteration of membrane rigidity and in the export process itself [37]. Some PHIST proteins contain DnaJ domains and might be involved in stabilization of exported proteins [35], they have been shown to be constituents of knobs [38], and recently have been found associated with exosomes [39]. Hence it is conceivable that PF3D7_0532300 might interact with SEMP1. PfEMP3 (PF3D7_0201900), a large PEXEL-containing protein, is expressed during the ring and trophozoite stage and associates with MCs. Subsequently it is further transported to the cytoplasmic face of the iRBC where it localizes to knobs but is also distributed more broadly [40]. PfEMP3 seems to be involved in PfEMP1 trafficking [41] and has been shown to bind actin and spectrin thus contributing to loss of mechanical stability of the RBC membrane [42], [43]. Whether SEMP1 is potentially involved in similar processes remains to be confirmed but identification in Co-IPs and the up-regulated expression of Pf332 in SEMP1-depleted parasites, although not significant, would support this notion because Pf332 has also been shown to modulate the RBC membrane skeleton [44].

SEMP1 belongs to the PEXEL-negative exported proteins, which includes proteins such as MAHRP1 [13], SBP1 [24], REX1 [7], and REX2 [10]. Modes of transport and targeting is still under debate and the most common feature is the necessity of the most N-terminal region. As with REX2 [10] and MAHRP2 [16] deletion of the first 16 amino acids completely abolished export of SEMP1 with the protein distributed throughout the parasite. Further N-terminal truncation of SEMP1 to the TM domain (SEMP1_72–123_-GFP) seem to have led to an arrest of transport within the ER which had also been observed when the MAHRP1 TM domain was fused to GFP [45]. In SEMP1, similar to other exported proteins [10], [45], [46], [47], the first 16 amino acids could be replaced either with the N-terminal SP of MSP1 or with the first 16 N-terminal residues of MAHRP2.

In conclusion, we identified and characterized a new MC protein termed SEMP1. Upon invasion it is exported early into the iRBC cytosol where it inserts into the MCs before it is at least partially translocated to the RBC membrane. A SEMP1 knock out parasite revealed that the protein is not essential for parasite survival, gametocytogenesis, or PfEMP1 export under culture conditions. Such lack of altered phenotypes after gene knock-out is not an exception but rather the rule. A large-scale study which previously produced knock-out parasites for 39 exported *P. falciparum* proteins only was able to identify phenotypical changes for 15 clones (38.5%) [37]. By using Co-IP and transcriptome analysis for functional characterization we provide a new starting point for further detailed dissection and characterisation of MC-associated proteins that show no phenotypical changes in knock-out parasites.

## Experimental Procedures

### Ethics statement

The animal work has been carried out according to relevant national guidelines. The immunization experiments in balb/c mice were approved by the Canton of Basel-Stadt (permit number: 2375). The manuscript has been prepared according to the Guidelines of ARRIVE (checklist S1).

### Cell culture

The *P. falciparum* 3D7 strain was cultured *in vitro* at 5% haematocrit as described [48] using RPMI medium containing 0.5% Albumax. Parasites were synchronized with 5% sorbitol as described [49].

### Plasmid constructs

Full-length *semp1* was PCR amplified using primers 5′-CAGTCTTAAGATGAGTCAACCACAAAAACAAC-3′ and 5′-CAGTATCGATTTTTGCGTTCTGTAAACTGGCT-3′ and cloned 5′ to *gfp* into pARL1mGFPmT [11] via AflII and ClaI restriction sites. PIESP2 was also cloned into pARL1mGFPmT via AflII and ClaI using primers 5′-ATTAACTTAAGATGTTACTCTTTTTTGCAAAAC-3′ and 5′-TAATTATCGATAGTTAGTAATAAATTATGAAGACC-3′. Truncated and mutated constructs for SEMP1 trafficking studies were generated similarly using primers summarized in table S1.

Primers 5′-GATCGGATCCTGAAAATGAGTCAACCACAAAAAC-3′ and 5′-GATCCCATGGTTTTGCGTTCTGTAAACTG-3′ were used to clone full-length SEMP1 5′ to 3xHA into pBcam-3xHA [50] via BamHI and NcoI restriction sites. Similarly PF3D7_0601900 was cloned into pBcam-3xHA via AflII and ClaI using primers 5′-CAGTCTTAAGATGACGGACCATTTATTGGATTT-3′ and 5′-CAGTATCGATATTTTCTGCATTGGCTGAAGCA-3′ and PF3D7_0702500 via BamHI and NotI using primers 5′- ATATGGATCCATGGCTTATCCTCTTTTAGAAGATG-3′ and 5′- ATATGCGGCCGCTACATGAGCTTCATTAGTGTTTAAAC-3′.

To disrupt the SEMP1 gene in 3D7 parasites, a DNA flank of approximately 500 bp starting 78 bp upstream of the SEMP1 start codon was inserted into the transfection vector pH-KO via PstI and EcoRI restriction sites using primers 5′-CAGTCTGCAGCTATTTTCCCTTTAACAATCTTTTT-3′ and 5′- CAGTGAATTCGACTTCATGAATTAATTATGCAATA-3′.

To fuse a FKBP destabilization domain (DD) to the C-terminus of the endogenous SEMP1 in 3D7 wild type parasites, an approximately 500 bp DNA flank of the 3′ end of SEMP1 was cloned into the transfection vector pARL-DD via BglII and AvrII restriction sites using primers 5′- CAGTAGATCTCCCAAGAAAAGAAATTCAACCC-3′ and 5′- CAGTCCTAGGTTTTGCGTTCTGTAAACTGGCT-3′.

### P. falciparum transfection

Ring stage 3D7 *P. falciparum* infected RBCs (parasitemia ∼10%) were transfected with 100 µg plasmid DNA as described [51] and cultured in the presence of either 10 nM WR99210 (pARL1mGFPmT / pH-KO / pARL-DD) or 2.5 mg/ml Blasticidin S (pBcam-3xHA). Drug resistant parasites were obtained approximately 4 weeks after transfection.

To select for integration of the SEMP1 flank into the parasite genome (pH-KO & pARL-DD) transfectants were cycled (3 weeks on /3 weeks off drug) three times and tested for integration by Southern blot. After integration of the SEMP1 flank into the endogenous gene was observed, a single parasite was isolated by serial dilution.

### Triton X-114 solubility assay

0.5 ml RBCs infected with 3D7 parasites episomally expressing SEMP1-3xHA were lysed on ice in 0.15% saponin. The pellet was resuspended in 200 µl 1% Triton X-114 in PBS containing protease inhibitors (Roche) and incubated on ice for 30 min. After centrifugation at 3000 g for 15 min, the supernatant was collected, incubated at 30 °C for 5 min and centrifuged at 20′000 g for 1 min. The supernatant was removed as the aqueous phase and the pellet was washed and resuspended with PBS to build the detergent phase. Equal amounts of both phases were analysed by Western blotting [52].

### Generation of SEMP1 mouse polyclonal antibodies

Codon-optimized (for expression in *E*. coli) full-length SEMP1 (Eurofins) was cloned 5′ to a 6xHis-tag into the expression vector pSCherry2 (Eurogentec), expressed in *E. coli* using the Cherry^TM^Codon kit (Eurgentec), and FPLC-purified with an anti-His column. Three mice each (female, balb/c, 16 weeks, Harlan Laboratories) were immunized intra-subcutanously or peritoneally with a 100 µl or a 200 µl dose respectively of SEMP1 (0.2 mg/ml) in PBS mixed with Sigma-Adjuvant-System (S6322, Sigma-Aldrich). Mice were boosted on day 21 and bled on day 31. All experiments were carried out at the Swiss Tropical and Public Health Institute (Basel, Switzerland) from November 2011 until December 2012, adhering to local and national guidelines and laws of experimental work with laboratory animals (Canton of Basel-Stadt permission no. 2375). Mice were randomly housed into 2 Macrolon cages of 3 mice each and individually marked. Mice were acclimatized for 2 weeks, hence at the onset of the experiment mice were 18 weeks old. Mice were kept under environmentally-controlled conditions (temperature, 22°C; relative humidity, 60–70%; light/dark cycle, 12/12 hours) and were fed on rodent pellets (Nafag; Gossau, Switzerland) and water ad libitum. At the end of the experiment mice were sacrificed using the standard CO_2_ inhalation method.

### Fluorescence microscopy

Thin smears of parasite infected red blood cells were fixed on glass slides with methanol at −20°C for 2 min, air-dried and blocked with 3% BSA in PBS. After incubation with primary antibodies mouse anti-SEMP1 serum (1∶100), mouse anti-GFP (Roche 1∶500), rabbit anti-GFP (Abcam 1∶200), rat anti-HA (Roche 1∶100), rabbit anti-MAHRP1 serum (1∶500) [16], rabbit anti-REX1 (1∶500) [53], mouse anti-SBP1 (1∶200) [22], mouse anti-PfEMP1 (1∶50) [25], mouse anti-Pf332 (1∶200) [54] and mouse anti-KAHRP (1∶500), slides were washed and incubated with Alexa Fluor 488/594 (Invitrogen 1∶200) conjugated goat anti-mouse antibodies, Alexa Fluor 488 (Invitrogen 1∶200) conjugated goat anti-rabbit antibodies or Alexa Fluor 568 (Invitrogen 1∶200) conjugated goat anti-rat antibodies. After washing, Vectashield Hard Set (Vector Laboratories) containing DAPI was added and covered by a glass coverslip. Images were obtained using a Leica DM 5000B fluorescence microscope. Images were analysed using Photoshop software.

Alternatively, SEMP1-3xHA infected red blood cells were fixed in 4% formaldehyde +0.01% glutaraldehyde in PBS. After washing in PBS the cells were permeabilized with 0.1% Triton X-100 in PBS and then blocked with 3% BSA in PBS. After incubation with primary antibodies rat anti-HA (Roche 1∶100) and rabbit anti-MAHRP1 serum (1∶500), cells were washed with PBS and incubated with Alexa Fluor 568 (Invitrogen 1∶200) conjugated goat anti-rat and Alexa Fluor 488 (Invitrogen 1∶200) conjugated goat anti-rabbit antibodies. After washing cells were resuspended in PBS, mixed with 0.4 volumes Vectashield Hard Set containing DAPI and mounted on a glass slide with a cover slip. Images were obtained using a Zeiss confocal microscope LSM 700.

### Electron microscopy

Immuno-electron microscopy of RBCs infected with 3D7 parasites episomally expressing SEMP1-GFP was performed according to Tokuyasu [55]. Cells were chemically fixed in 0.1 M phosphate buffer containing 2% paraformaldehyde and 0.2% glutaraldehyde, embedded in gelatine and cryo-preserved in 2.3 M sucrose. Gelatine blocks were frozen in liquid nitrogen and sectioned at −120°C using an ultra-microtome (UC7, Leica) to generate 70–80 nm sections. Immuno-labelling was done in 1% BSA in PBS with rabbit anti-GFP primary antibodies (Abcam, 1∶40) and 5 nm immunogold-coupled Protein A (CMC Utrecht, 1∶70). After immune-labelling, the sections were stained at 4°C in a 1∶9 mixture of 4% uranyl acetate and 2% methylcellulose. Images were taken with a CM100 at 80 kV.

### Western blot analysis

0.5 ml infected red blood cells were lysed on ice in 0.15% saponin in PBS. The resulting parasite pellet was resuspended in Laemmli sample buffer, separated on a 12.5% acrylamide gel or a NuPAGE 4–12% Bis-Tris gel (Novex) and blotted to nitrocellulose (Hybond-C extra; GE Healthcare) using a Trans-Blot semi-dry electroblotter (Bio-Rad). The membrane was blocked in 10% skim milk +0.1% Tween in Tris-buffer. The used primary antibodies were mouse anti-SEMP1 serum (1∶1000), mouse anti-HA (Roche 1∶5000), mouse anti-GAPDH (1∶20′000) [56], mouse anti-GFP (Roche 1∶1000) or rabbit anti-MAHRP2 serum (1∶2000) [16]. The used secondary antibodies were horseradish peroxidase-conjugated goat anti-mouse (Pierce, 1∶20 000) and goat anti-rabbit (Acris, 1∶5000) IgG.

### Southern blot analysis

Genomic DNA was isolated by phenol/chloroform extraction of saponin lysed parasites as described [57]. DNA was digested with either ClaI & XmaI (SEMP1-KO) or EcoRI (SEMP1-DD) restriction enzymes, separated on a 0.8% agarose gel and transferred onto a Amersham Hybond-N^+^ membrane (GE Healthcare). Blots were probed with ^32^P-dATP-labelled h*dhfr* PCR fragments.

### Co-Immunoprecipitation

240 ml culture (5% hematocrit, 8% parasitemia) of 3D7 parasites episomally expressing SEMP1-3xHA was cross-linked in 1% formaldehyde at 37°C. Reaction was stopped after 10 min by addition of 0.125 M glycine and transfer on ice. Cells were pelleted at 700 g for 5 min, washed in ice cold PBS and red blood cells lysed on ice in 0.15% saponin. After additional washing in PBS the pellet was resuspended in 1.5 ml sonication buffer (50 mM Tris-HCl pH 8.0, 10 mM EDTA, 1% SDS) containing complete protease inhibitor cocktail and sonicated with a Bioruptor UCD-300 for 10 min (30 sec on /30 sec off). After centrifugation at 20′000 g for 10 min the supernatant was collected. 650 µl supernatant was mixed with 650 µl 2× binding buffer (50 mM Tris-HCl pH 7.4, 300 mM NaCl, 2% NP40) and for the negative control additionally 150 µg HA peptide (Sigma) was added. Samples were mixed with 150 µl α-HA affinity matrix (Roche) and incubated at 4°C for 16 h at 25 rpm. The beads were pelleted at 300 g for 1 min and the supernatant removed and collected (supernatant). After washing for 4×5 min with 1 ml washing buffer (50 mM Tris-HCl pH 7.4, 150 mM NaCl, 1 mM EDTA, 1% NP40) the final supernatant was collected (wash) and the beads mixed with 40 µl elution buffer (1× binding buffer containing 0.5 mg/ml HA peptide and protease inhibitors). After incubation at 25 rpm for 2 h the beads were pelleted at 300 g for 1 min and the supernatant collected (elution). The protein content of the elution was subsequently analyzed by mass-spectrometry from either TCA precipitated pellets or from extracted gel bands after separation on a NuPage 4–12% Bis-Tris gel (Novex) and Coomassie staining.

### Comparative transcriptome analysis by microarray

After sorbitol-synchronization to an 8 h window, a culture of SEMP1-DD parasites was split and cultured in the presence or absence of Shield. At four specific time points (6–14 hpi, 16–24 hpi, 26–34 hpi & 36–44 hpi) parasite RNA was isolated from 30 ml culture (5% hematocrit, 5% parasitemia) using RiboZol RNA extraction reagent (Amresco). RNA was transcribed into cRNA and Cyanine 5-labelled by incorporation of Cy5-CTP using the Quick Amp labelling kit (Agilent). 450 ng Cy5-labelled SEMP1-DD cRNA containing 5 pmol dye were mixed with an equal amount of Cy3-labelled 3D7 reference cRNA generated from mixed stages (equal amounts of ring-, trophozoite- and schizont-stage RNA) and hybridized onto an Agilent *P. falciparum* microarray (AMADID #037237) as described [19]. After washing, the slide was air-dried and scanned using a GenePix 4000B microarray scanner and GenePix Pro 7 software. Lowess normalization and background elimination was performed using Acuity 4.0 software (Molecular Devices). SEMP1-dependent transcriptional changes were identified by either Significance Analysis of Microarrays (SAM) [20] or by Student's t-test. Heatmap of candidate genes was generated using Cluster and TreeView software [58].

### CD36 binding assay

Recombinant human CD36 (125 µg/ml in PBS) or 1% BSA in PBS (control) was immobilized on a Petri dish as described [59]. After incubation in a humid chamber over night at 4°C, unspecific binding was blocked with 1% BSA in PBS for 30 min at room temperature and washed with RPMI-Hepes. Infected RBCs in RPMI-Hepes and 10% serum (5% hematocrit) were added and incubated for 30 min at 37°C. After gentle washing with RPMI-Hepes, bound cells were fixed with 2% glutaraldehyde in RPMI-Hepes for 2 h and stained with 10% Giemsa for 10 min. Bound infected RBCs were quantified in 8 or 10 different 0.2 mm^2^ areas. The mean value was calculated as infected RBCs per mm^2^ and normalized to a parasitemia of 1%. The experiment was performed three times.

### Induction of gametocytogenesis

A 10 ml SEMP1-KO parasite culture (5% hematocrit, 6% parasitemia) was sorbitol-synchronized and cultured for 24 h in 20 ml RPMI medium containing 1% Albumax. After dilution to a parasitemia of ∼2% trophozoites, to prevent overgrowth, the committed parasites were cultured in 10 ml normal RPMI medium (0.5% Albumax) for additional 24 h. Then parasites were cultured for 4 days in 10 ml medium containing 50 mM N-Acetylglucosamine (GlcNac) (replaced daily).

## Supporting Information

Figure S1 **Recombinant expression of potential interaction partners.** A: Lysates of 3D7 parasites expressing PF3D7_0702500-3xHA (0702500-3xHA), PF3D7_0601900-3xHA (0601900-3xHA) and PIESP2-GFP generated by saponin lysis and analysed by Western blot using rat α-HA / mouse α-GFP antibodies.
(TIF)

Table S1 **Primers used to generate truncated and mutated constructs for SEMP1 trafficking studies.** *Afl*II and *Cla*I restriction sites (RS) thereby allowed directional cloning into the pARL1mGFPmT transfection vector.
(DOCX)

Checklist S1 **ARRIVE Checklist.**
(ASP)
