# Supplementary figures and images for: Characterization of the Small Exported Plasmodium falciparum Membrane Protein SEMP1
Source: PLoS One. 2014 Jul 25;9(7):e103272. doi: 10.1371/journal.pone.0103272 (PMC4111544; doi:10.1371/journal.pone.0103272)

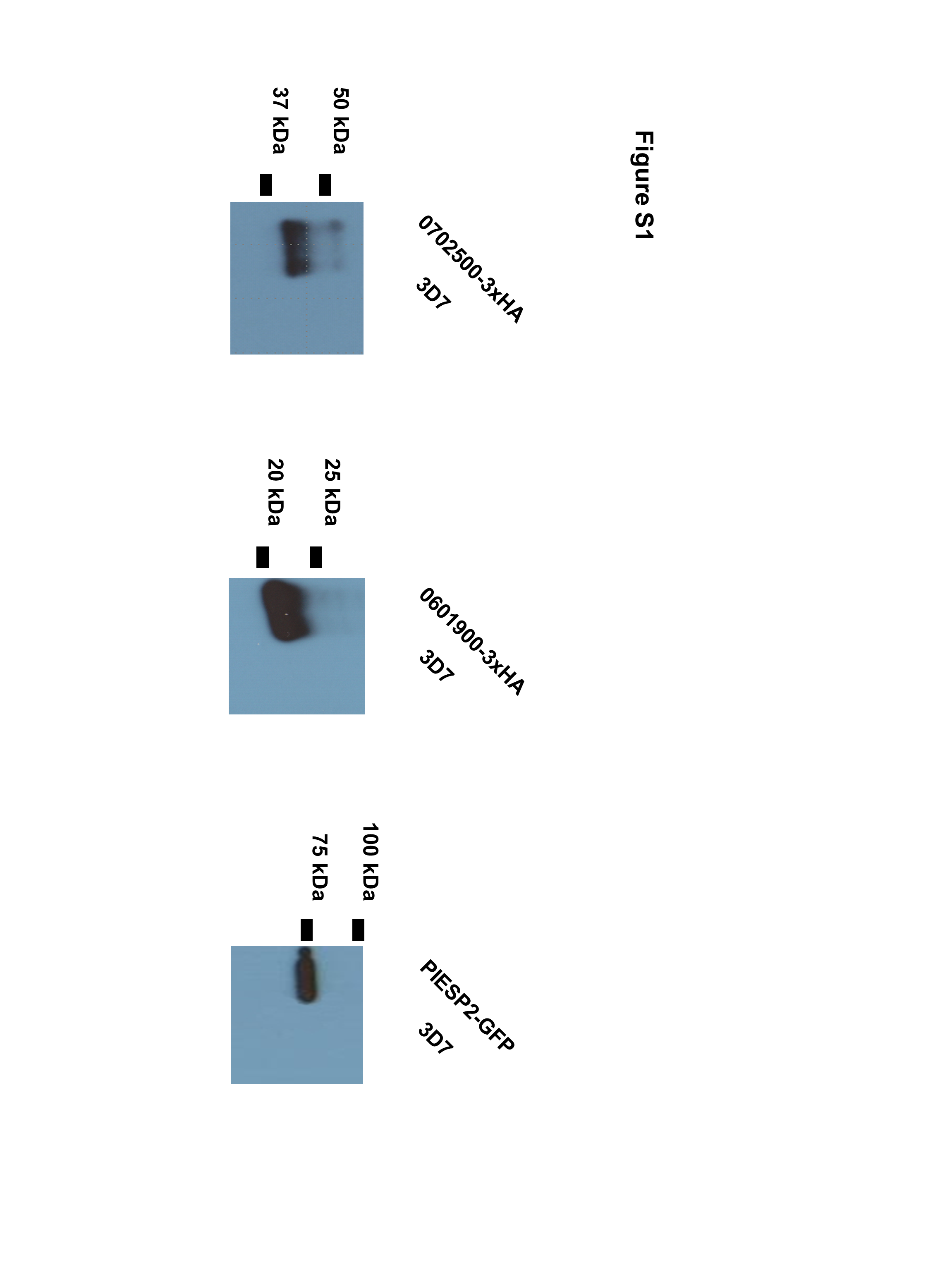

Supplement: Figure S1 — Recombinant expression of potential interaction partners. A: Lysates of 3D7 parasites expressing PF3D7_0702500-3xHA (0702500-3xHA), PF3D7_0601900-3xHA (0601900-3xHA) and PIESP2-GFP generated by saponin lysis and analysed by Western blot using rat α-HA / mouse α-GFP antibodies. (TIF) [file pone.0103272.s001.tif]
