# Supplementary material for: Characterization of the Small Exported Plasmodium falciparum Membrane Protein SEMP1
Source: PLoS One. 2014 Jul 25;9(7):e103272. doi: 10.1371/journal.pone.0103272 (PMC4111544; doi:10.1371/journal.pone.0103272)
Supplement: Table S1 — Primers used to generate truncated and mutated constructs for SEMP1 trafficking studies. AflII and ClaI restriction sites (RS) thereby allowed directional cloning into the pARL1mGFPmT transfection vector. (DOCX) [file pone.0103272.s002.docx]

| **Construct** | **Primer name** | **Primer sequence** | **RS** |
| --- | --- | --- | --- |
| SEMP1_17-123_-GFP | 17-123-F | CAGTCTTAAGATGGCCAATACCCAAGAAAAGAAATT | AflII |
|  | 17-123-R | CAGTATCGATTTTTGCGTTCTGTAAACTGGCT | ClaI |
| SEMP1_72-123_-GFP | 72-123-F | CAGTCTTAAGATGGAGTTGGTTGAATTTGGTTTAAAC | AflII |
|  | 72-123-R | CAGTATCGATTTTTGCGTTCTGTAAACTGGCT | ClaI |
| SEMP1_1-97_-GFP | 1-97-F | CAGTCTTAAGATGAGTCAACCACAAAAACAAC | AflII |
|  | 1-97-R | CAGTATCGATTACGTAATCATATATTTGTAAGGC | ClaI |
| MSP_1-16_SEMP1_17-123_-GFP | MSP1-F | CAGTCTTAAGATGAAGATCATATTCTTTTTATGTTC  ATTTCTTTTTTTTATTATAAATACACAATGTGTAG  CCAATACCCAAGAAAAGAAATT | AflII |
|  | MSP1-R | CAGTATCGATTTTTGCGTTCTGTAAACTGGCT | ClaI |
| M2_1-16_SEMP1_17-123_-GFP | M2-F | CAGTCTTAAGATGCAGCCTTGTCCATATGATGTATA  CAATCAAATAAACCATGTAGGAACTCATTGGGCTGC  CAATACCCAAGAAAAGAAATT | AflII |
|  | M2-R | CAGTATCGATTTTTGCGTTCTGTAAACTGGCT | ClaI |

**Table S1.** Primers used to generate truncated and mutated constructs for SEMP1 trafficking studies. *Afl*II and *Cla*I restriction sites (RS) thereby allowed directional cloning into the pARL1mGFPmT transfection vector.
